# How fish traits and functional diversity respond to environmental changes and species invasion in the largest river in Southeastern China

**DOI:** 10.7717/peerj.11824

**Published:** 2021-07-23

**Authors:** Li Lin, Weide Deng, Xiaoxia Huang, Yang Liu, Liangliang Huang, Bin Kang

**Affiliations:** 1 College of Fisheries, Ocean University of China, Qingdao, Shandong, China; 2 Henry Fok College of Biology and Agriculture, Shaoguan University, Shaoguan, Guangdong, China; 3 Department of Oceanography, National Sun Yat-Sen University, Kaohsiung, Taiwan, China; 4 Key Laboratory of Atmospheric Environment and Processes in the Boundary Layer Over the Low-Latitude Plateau Region, School of Earth Science, Yunnan University, Kunming, Yunnan, China; 5 College of Environmental Science and Engineering, Guilin University of Technology, Guilin, Guangxi, China; 6 Key Laboratory of Mariculture (Ocean University of China), Ministry of Education, Qingdao, Shandong, China

**Keywords:** The Min River, Fish fauna, Functional diversity, Turnover, Environmental filtering, Species interaction

## Abstract

**Background:**

Freshwater fish populations are facing multiple stressors, including climate change, species invasion, and anthropogenic interference. Temporal studies of fish functional diversity and community assembly rules based on trait-environment relationships provide insights into fish community structure in riverine ecosystems.

**Methods:**

Fish samples were collected in 2015 in the Min River, the largest freshwater riverine system in Southeastern China. Fish functional diversity was compared with the background investigation in 1979. Changes in functional richness, functional evenness, functional divergence, and functional beta diversity were analyzed. Relationships between functional diversity and environmental factors were modeled by random forest regression. Correlations between fish functional traits and environmental factors were detected by fourth-corner combined with RLQ analysis.

**Results:**

Functional richness was significantly reduced in 2015 compared with 1979. Functional beta diversity in 2015 was significantly higher than that in 1979, with functional nestedness being the driving component. Reduction of functional richness and domination of functional nestedness is associated with species loss. Trait convergence was the dominant mechanism driving the temporal changes of functional diversity. Precipitation, temperature, species invasion, and human population were the most significant factors driving fish functional diversity. Higher precipitation, higher temperature, and presence of invasive species were significantly associated with higher swimming factor and higher relative eye diameter, while the opposite environmental conditions were significantly associated with higher pectoral fin length and eurytopic water flow preference.

**Conclusions:**

Environmental filtering is the dominant temporal assembly mechanism shaping fish community structure. This work contributes to the understanding of temporal freshwater fish community assembly and the associations between fish functional structure and local environmental conditions, which will be informative for future freshwater fish conservation.

## Introduction

Assembly rule theory proposes that local communities are formed as species go through a series of hierarchical filters from the regional species pool, including dispersal, abiotic and biotic factors (Keddy, 1992). First, dispersal defines the geographical distance at which a species can stablish (Zha et al., 2020). Traits related to dispersal capabilities may determine which species reach and colonize a given area, and which species succeed in securing adequate resources preemptively. Second, environmental filtering selects species according to their preference and tolerance to local abiotic conditions (Craven et al., 2018). It determines species presence or absence according to their environmental niche. Retained species tend to share similar traits, resulting in trait clustering (Kraft et al., 2015). Third, species interact with each other through negative or positive biotic interactions (Che et al., 2019). It determines relative abundance of a species in a given set of biotic conditions. Environmental filtering selects species with similar functional traits and results in trait convergence (Craven et al., 2018). Biotic filtering, especially interspecific competition caused by exotic species, exclude species with similar traits because of common resources utilization and result in trait divergence (Suarez-Castro et al., 2020).

Functional diversity (FD) is a useful tool to test assembly rules (Che et al., 2019; Liu & Wang, 2018; Mouchet et al., 2010). Functional richness (FRic), functional evenness (FEve), and functional divergence (FDiv) are three primary components of functional alpha diversity (Mason et al., 2005). FRic measures the size of the functional space extracted from functional traits, FEve measures the regularity of functional traits distributed in the functional space, and FDiv defines how far species are from the center of the functional space (Villeger, Mason & Mouillot, 2008). These three facets of FD are complementary to each other and useful in detecting relationships between organisms and environments (Schumm et al., 2019), explaining spatiotemporal variations in community structures (Garcia-Navas et al., 2020), and identifying critical ecological risks under abiotic or biotic stresses (Ramirez-Ortiz et al., 2020; Yao et al., 2021). Functional beta diversity measures functional dissimilarity. Patterns of functional beta diversity may capture important signals of niche-based assembly processes that are not evident from patterns of taxonomic beta diversity (Swenson, 2014). For example, environmental filtering could be detected by low functional beta diversity between two communities because of increased trait similarity, but they possibly still exhibit high taxonomic beta diversity (Villeger et al., 2012).

Physical and chemical conditions such as nutrient concentration, temperature, precipitation, and pH are strongly associated with fish habitat suitability and influence fish community structures (Wijewardene et al., 2021). Anthropogenic interferences such as overfishing, urbanization, and dam construction, and interspecific interactions such as predation, competition, and facilitation are also widely recognized as important factors affecting species spatiotemporal patterns (Che et al., 2019; Dolbeth et al., 2016). Previous studies have shown the usefulness of functional traits in elucidating assembly rules in freshwater fish communities, which has improved the understanding of mechanisms behind freshwater fish community structure (Zha et al., 2020). For example, the research in temperate streams across America revealed that fish community assembly was governed by environmental filtering rather than interspecific competitions (Giam & Olden, 2016). The study on European and Amazonian stream fishes suggested that French stream fish communities were mainly under strong environmental filtering, whereas dispersal limitation was the predominant process in structuring Guianese stream fish communities (Cilleros et al., 2016). However, most of these studies were spatially scaled, and the questions that how fish FD changes over time and which process governs freshwater fish community temporally need to be better explained. Riverine fish community assembly at temporal scales has not been well illuminated because temporal data are scarcer than spatial data, which compromises our understanding of temporal patterns in riverine fish communities. Temporal study on community assembly contributes to predictive community ecology and conservation biology (Rull, 2012). For instance, disentangling driving factors of community assembly will help select important variables for predicting species redistribution under climate change (Zakharova, Meyer & Seifan, 2019), and systematic conservation planning needs information on historical changes of community to prioritize species and areas of conservation interest (Kirkman et al., 2019).

The Min River is the largest basin in Southeastern China. Several invasive species have been identified in this river (Deng et al., 2020). Human interferences, such as urbanization, dam construction, and agriculture, have inevitably changed its ecological environments (Ma et al., 2020; Ren et al., 2016; Zhao et al., 2016). Temperature and precipitation are continuously changing in the basin (Deng et al., 2020). Also, its dendritic spatial structure might be intrinsically correlated with fish dispersal (Perkin & Gido, 2012). Historical fish investigation in this river can be traced back to 1979, which found 125 species, 99 genera, 31 families, and 14 orders (Lian, 1988). By sampling at the same sites with comparable efforts with this historical investigation, studies on fish communities can help establish how fish communities changed over decades. All these prerequisites make the Min River an ideal study system to discuss freshwater fish community assembly at temporal scales.

A temporal decrease in functional richness was expected in the Min River, but we did not presume how functional evenness, functional divergence, or functional dissimilarity changed since 1979. We hypothesized that both environmental and biotic filtering were the main temporal community assembly rules, but we did not clearly anticipate which process was dominant and which factors were the most important in driving these processes. Different responses of fish functional traits to environmental factors were also expected, but we did not preconceive which traits were significantly correlated with those environmental factors. Thus, by sampling in the Min River in 2015 at the same sites with equivalent efforts of 1979, we aimed to answer the following questions: (1) How does fish FD change temporally, including functional alpha and beta diversity, and which process, trait convergence or divergence, governs these temporal changes? (2) Which environmental factors are the most important for these assembly processes? (3) How do fish functional traits correlate with these factors? We expected a better understanding of temporal community assembly in riverine ecosystems. To our best knowledge, this is one of the first times that try to disentangle temporal freshwater fish community assembly mechanisms in subtropical areas in China.

## Materials and Methods

### Study area and field sampling

**Figure 1:**
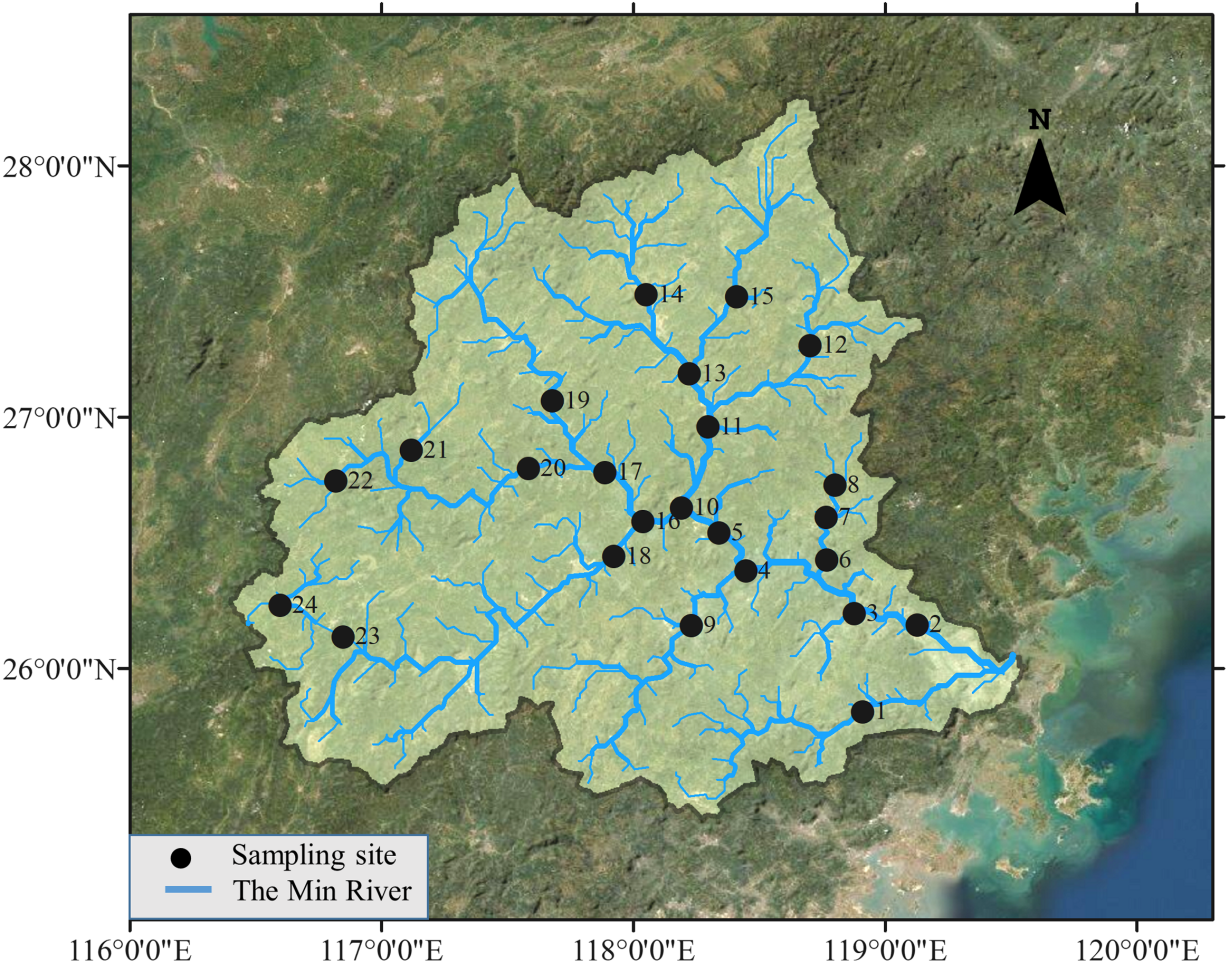
Study area and sampling sites. Fish samples are collected in 24 sites distributed along the Min River, the largest river in Southeastern China.

The Min River is located in Southeastern China, ranging from 116.38°E to 119.72°E and 25.38°N to 28.32°N. The river runs through Fujian Province with a length of 584 km and a basin area of about 61,000 km^2^. Both sampling in 1979 and 2015 were conducted by traditional gill nets fishing (20 × 2.5 m, 3 layers with mesh sizes of 1, 2, and 3 cm) at the same 24 sites in May from the upstream to the lower reach of the river (Fig. 1). Three gill nets were placed in the left, middle, and right parts of the river at each site. The lead line settled the net on the river bottom, and the float line kept it vertical in the water column, allowing it to capture fish as they swim. Gill nets were set before sunset and lifted after sunrise to cover maximal peaks of fish activity. According to the experts who participated in the sampling in 1979, electrofishing was also used in 1979, but detailed information was not documented. As suggested by these experts, additional electrofishing (backpack electrofishing unit, Model: CWB-2000 P, Yufengda, China; 12-V import, 250-V export) which lasted 30 min and extended 50 m along the river course was applied in 2015 in wading areas at each site to assure that sampling effort in 2015 was at least as exhaustive as that in 1979. Species in 1979 and 2015 were identified and validated according to FishBase to avoid invalid species, synonyms, and homonyms (Froese & Pauly, 2019).

Permission to conduct the study was granted by the Animal Care and Ethics Committee of Ocean University of China (No. 20150305003). Field experiments in 2015 were approved by Research Council of Ocean University of China (No. 20150305004).

### Environmental variables

We considered eight environmental variables, covering climatic variables (temperature and precipitation), human factor (human population), geographic variables (altitude and slope), river structure variables (Strahler order of stream segment and downstream link), and presence or absence of species invasion. Temperature (°C) and precipitation (mm) in 1979 and 2015 were derived from “China surface climatological data set (V3.0)” downloaded from China meteorological data service network (https://data.cma.cn/). Geostatistical spatial analyses were used to generate the inverse distance weight interpolation (IDW) map and describe the spatial variability in the mean annual temperature and precipitation at each site (Lu & Wong, 2008). Human population (thousand) at the county scale in 1979 and 2015 was derived from the China Population Statistics Yearbook (National Bureau of Statistics, 1980) and (National Bureau of Statistics, 2016). Altitude (m) and slope at 30 arc-second resolution were extracted from EarthEnv (https://www.earthenv.org/topography) (Amatulli et al., 2018). Two categorical variables were applied as the surrogates for spatial river structure, including Strahler order of stream segment (Order) (Strahler, 1957) and downstream link (Dlink) (Osborne & MJ, 1992). For Dlink variable, the magnitude of a link is firstly defined, which is the number of first-order segments upstream of a given point on a channel, then the Dlink at any point is the magnitude of the link below the next downstream confluence at any point. Species invasion was represented by a binary factor, where “1” refers to presence of non-native species, and “0” refers to absence of such species.

### Measurement of functional traits

Ten morphological and ecological traits were selected for FD calculation based on two reasons: (1) these traits are associated with different functional groups, including feeding habit, trophic, swimming capability, habitat preference, and life cycle of the fish; and (2) these traits capture the response of fish species to abiotic and biotic environments, i.e., they are response traits (Violle et al., 2007). Morphological traits were relative head length, relative eye size, relative snout length, relative head depth, relative pectoral fin length, swimming factor, and relative body depth, while ecological traits were water flow preference, position in the water column, and maximum life span (Table 1) (Gatz Jr, 1979; Pool, Grenouillet & Villeger, 2014; Toussaint et al., 2016; Villeger, Grenouillet & Brosse, 2013; Watson & Balon, 1984).

**Table 1:** Ten functional traits used in functional diversity analysis and their ecological meanings.

**Functional trait**	**Type**	**Formula(Code)**	**Ecological meaning**
Relative head length	Morphological	Hl/Sl	High values may indicate fish able to feed on relatively larger prey (Watson & Balon, 1984).
Relative eye size	Morphological	Ed/Hd	Visual acuity, relating to prey detection (Gatz Jr, 1979).
Relative snout length	Morphological	Snl/Hl	The length of the snout affects a variety of trophic and sensory capabilities, influencing the abilities of fishes to detect and acquire prey (Toussaint et al., 2016).
Relative head depth	Morphological	Hd/Bd	High values indicate deeper heads. Head depth plays a variety of roles in the sensory and trophic capabilities of a fish. Deep heads also affect the hydrodynamics of a fish, increasing maneuverability (Toussaint et al., 2016).
Relative pectoral fin length	Morphological	PecFl/Sl	Pectoral fin length is assumed to increase as a function of the amount of low-speed maneuvering in the behavior of fish (Watson & Balon, 1984).
Swimming factor	Morphological	CPd/CFd	Hydrodynamics. Caudal propulsion efficiency through reduction of drag (Villeger, Grenouillet & Brosse, 2013).
Relative body depth	Morphological	Bd/Sl	Relative body depth is assumed to be inversely related to habitat water velocity and directly related to the capacity of making vertical turns (Gatz Jr, 1979).
Water flow preference	Ecological	Rheophilic; Limnophilic; Eurytopic.	Prefer fast flows (rheophilic), slow flows (limnophilic), or be adapted for a wide range of flow types (eurytopic) (Pool, Grenouillet & Villeger, 2014).
Position in the water column	Ecological	Benthic; Benthopelagic; Pelagic.	Preference to the vertical water level. Prefer bottom of the water (Benthic), middle water level (Benthopelagic), or surface water (Pelagic) (Pool, Grenouillet & Villeger, 2014).
Maximum life span	Ecological	Life<10; Life≥10, <20; Life≥20.	Potential longevity below 10 years (Life<10), between 10 and 20 years (Life≥10, <20), or above 20 years (Life≥20) (Pool, Grenouillet & Villeger, 2014).

**Notes.**

Sl standard length Hl head length PecFl pectoral fin length Snl snout length Bd body depth Hd head depth Ed eye diameter CPd caudal peduncle depth CFd caudal fin depth

Measurements of these characteristics are shown in Fig. S1.

Morphological traits were derived from basic morphological measurements, including standard length, head length, pectoral fin length, snout length, body depth, head depth, eye diameter, caudal peduncle depth, and caudal fin depth (Fig. S1). To mitigate the influence of intraspecific trait variations, at least 30 individuals of each species were required in both 1979 and 2015, and for those species with less than 30, all samples were measured. Samples subjected to measurements were individuals of similar size for each species, which also exceeded the minimum length of sexual maturity extracted from FishBase (Froese & Pauly, 2019) and “The Fish of Fujian Province” (Zhu, 1984). To mitigate the impact of deformations caused by dehydration in preserved samples on the accuracy of data in 1979, only intact individuals were measured and values of morphological traits for calculating FD were ratios of these basic morphological measurements. Ecological traits were extracted from FishBase (Froese & Pauly, 2019) and other ichthyological literatures (Chen, 1998; Chu, Zhen & Dai, 1999; Wu & Zhong, 2008; Yue, 2000; Zhu, 1984).

### Functional diversity metrics

FRic, FEve, and FDiv were used because they measure size, regularity, and divergence of species in the functional space, respectively (Laliberté & Legendre, 2010; Villeger, Mason & Mouillot, 2008). Values of FEve and FDiv vary from 0 to 1, while values of FRic had no limit. Sørensen dissimilarity (FDsor) was used to calculate functional beta diversity (Villeger, Novack-Gottshall & Mouillot, 2011). It is decomposed into two components, turnover (FDsim) and nestedness (FDsne) (Villeger, Grenouillet & Brosse, 2013). By assuming that fish morphological traits in the Min River did not evolve at the temporal scale of decades, trait matrices in 1979 and 2015 were combined into a single matrix with mean morphological trait values of species. Then, this single matrix was subjected to Principal Coordinate Analysis (PCoA) with Gower’s distance using R package “ade4” (Anderson & Willis, 2003; Gower, 1966). Because FRic and FDiv both require more species than traits (Laliberté & Legendre, 2010) and the smallest species richness in this work was six, the first five PCoA axes, which explained 82.64% of cumulative variances, were preserved for FD calculations. Finally, R package “FD” was used to calculate FRic, FEve, and FDiv (Laliberté & Legendre, 2010), and “betapart” to calculate FDsor, FDsim, and FDsne (Baselga et al., 2020).

Paired *t*-test was used to test the difference of species richness, FRic, FEve, FDiv, FDsor, FDsim, and FDsne between 1979 and 2015. When paired *t*-test detected no significant difference, Levene’s test was used to test the difference of variance (Fox & Weisberg, 2019).

### Random forest regression

Random forest (RF) regression was applied to explore the impacts of environmental factors on FRic, FEve, FDiv, FDsor, FDsim, and FDsne given that RF does not require a priori determination of probability density distribution (Breiman, 2001). Variance inflation factor (VIF) was applied to test multicollinearity among environmental factors with a stepwise procedure (Dormann et al., 2013). All selected environmental factors had a VIF below 5. Response variables in RF were resampled 5000 times to generate a large number of decision trees. The importance of an environmental factor to the model was calculated as how much the mean standard error (MSE) increased when this variable was permuted. The more the MSE increased, the more important this variable was. Ten repeats of five-fold cross-validation were applied to check the performance of the model, then the most important variables which yielded the least cross-validation error were identified. Partial dependence plots were used to visualize the response of FD to these important variables, which were classified into five categories: positive, negative, unimodal, bimodal, and irregular (Goldstein et al., 2015). R package “randomForest” was used for modeling (Liaw & Wiener, 2002), “usdm” for stepwise VIF testing (Naimi et al., 2014), and “pdp” for partial dependence plotting (Brandon, 2017).

### Fourth-corner combined with RLQ

The association between functional traits and environmental variables was tested using the fourth-corner combined with RLQ approaches (Dray et al., 2014; Dray & Legendre, 2008). Species occurrence matrix (**L**) was ordinated by correspondence analysis (CA). Environmental variables matrix (**R**) was ordinated by Hill-Smith analysis for a mix of qualitative and quantitative variables (Hill & Smith, 1976). Traits matrix (**Q**) was ordinated by PCoA analysis with Gower’s distance as was used in FD calculation. Fourth-corner analysis was performed to correlate fish functional traits and environmental variables with the first two axes of RLQ. Following the proposal of Dray et al. (2014), this analysis combined permutation models 2 and 4 and used 9,999 repetitions to test the null hypothesis (H_0_) that the species traits (**Q**) are not related to the environmental variables (**R**). In permutation model 2, sites were randomized to test the relationship between species occurrence (**L**) and the environmental variables (**R**), and in permutation model 4, species were randomized to test the relationship between species occurrence (**L**) and their traits (**Q**) (Dray & Legendre, 2008). H_0_ is rejected when significant relationships are found in both permutation models. *P* values were adjusted using the false discovery rate (FDR) procedure to mitigate type I error (Benjamini & Hochberg, 1995). The R package “ade4” was used for the fourth-corner combined with RLQ analysis (Dray & Dufour, 2007).

## Results

### Temporal changes of functional diversity

**Figure 2:**
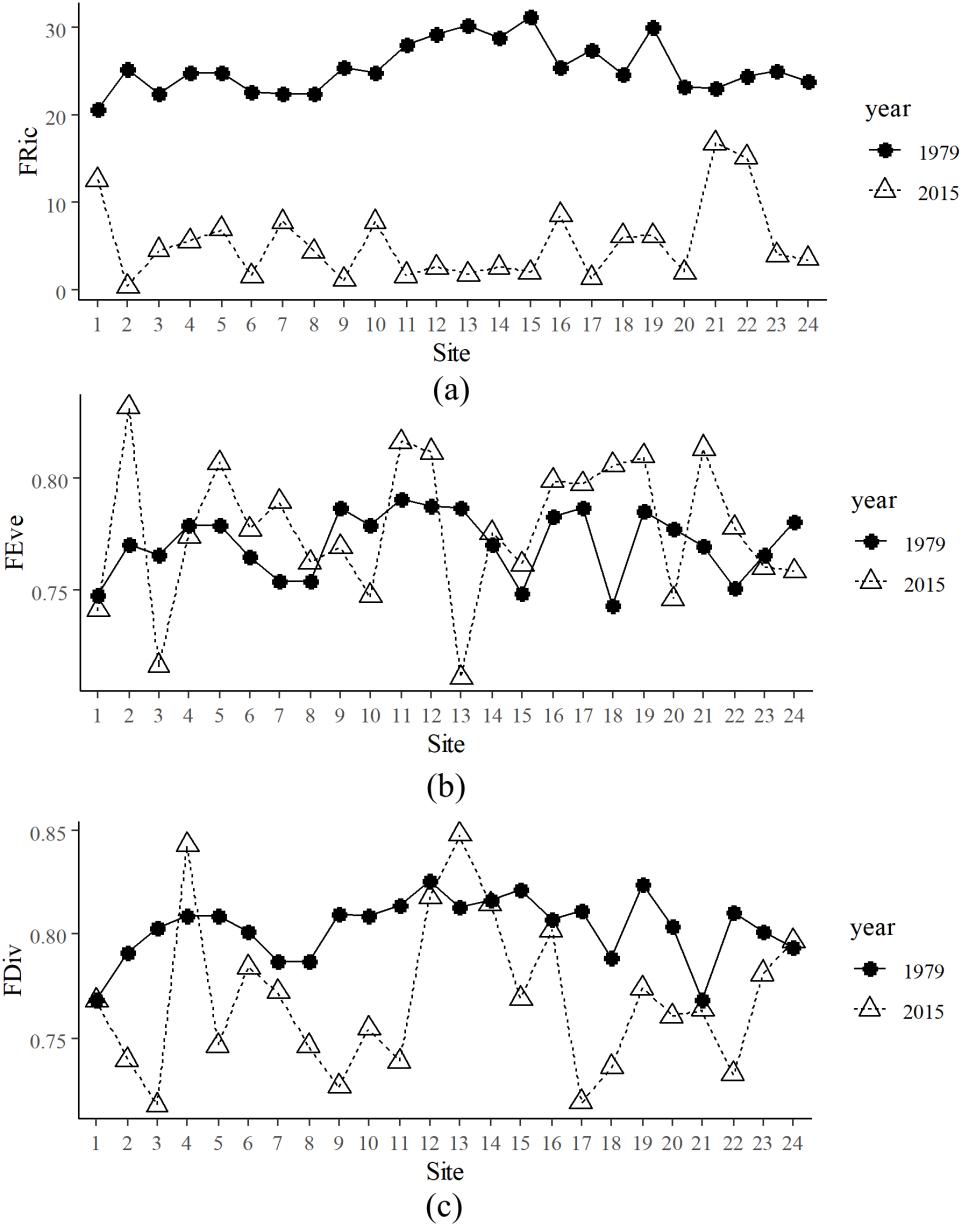
Spatial and temporal functional alpha diversity in the Min River. (A) Functional richness (FRic), (B) functional evenness (FEve), and (C) functional divergence (FDiv) at various sites in 1979 and 2015.

There were 125 species in 1979 and 82 species collected in 2015, 56 of which were shared by both periods. Species richness in 1979 was significantly higher than in 2015 (Table S1). FRic in 1979 was significantly higher than that of 2015 (Fig. 2A). Mean FRic in 1979 was 25.32 (± 3.35 SE), with the highest value at site 15, while mean FRic in 2015 was 7.00 (± 5.29 SE), with the highest value at site 21. FEve in 2015 was not significantly different from that of 1979, but Levene’s Test for equality of variances confirmed that variance of FEve in 2015 was significantly higher than that in 1979 (Fig. 2B). Paired *t*-test confirmed that FDiv in 1979 was significantly higher than that in 2015, except that FDiv at sites 4 and 13 were lower in 1979 than that in 2015 (Fig. 2C).

**Figure 3:**
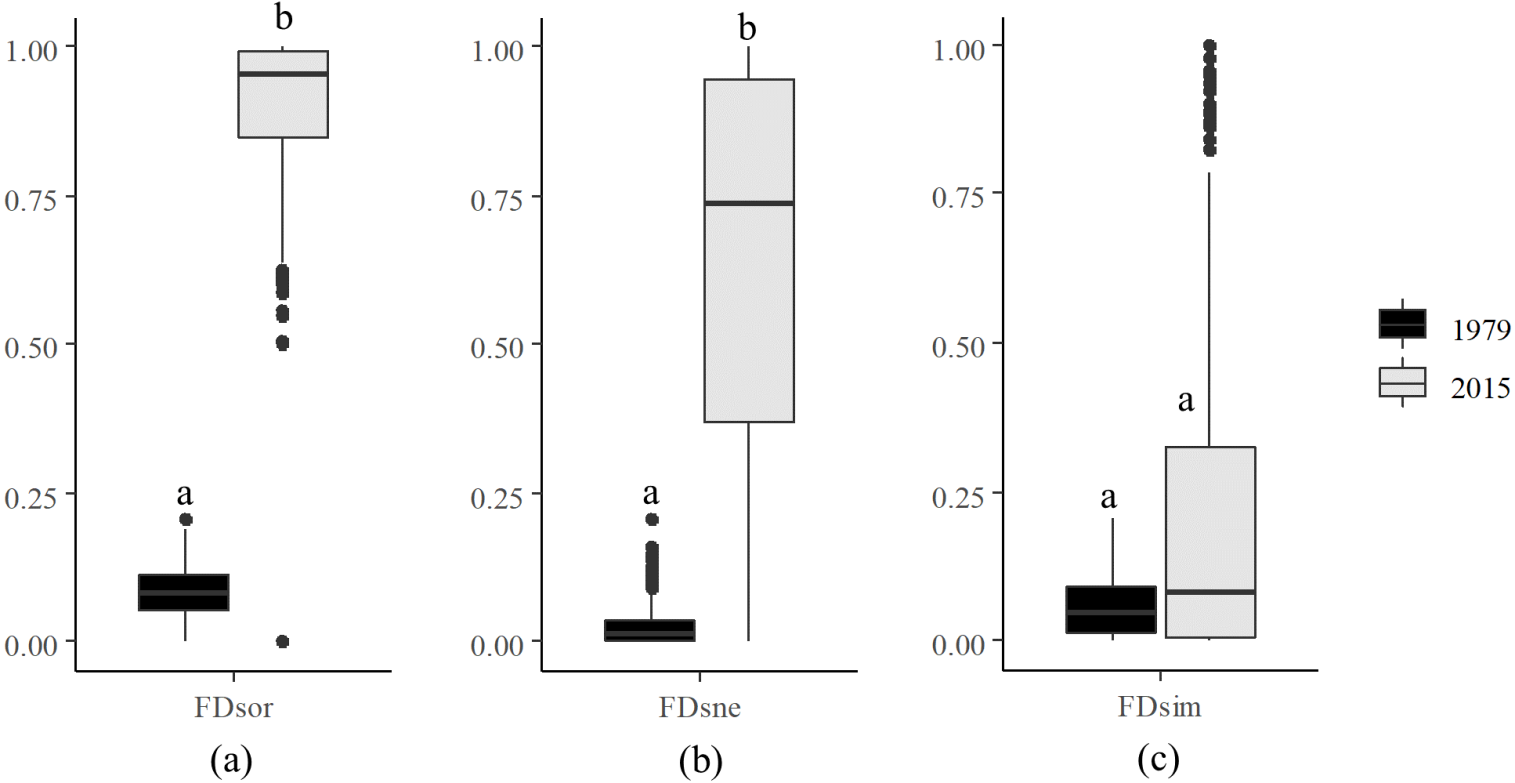
Decomposition of functional beta diversity. (A) Total functional beta diversity (FDsor), (B) nestedness (FDsne), and (C) turnover (FDsim) in 1979 and 2015. Different letters above the boxes indicate significant differences in FDsor, FDsne, and FDsim between 1979 and 2015, respectively (paired *t*-test, *p* < 0.001).

Decomposition of functional beta diversity revealed the main component driving FD dissimilarities in the river. FDsor (Fig. 3A) and FDsne (Fig. 3B) in 2015 were significantly higher than those in 1979, respectively, but FDsim (Fig. 3C) in 2015 was not statistically different from that in 1979. These results indicate that difference in FDsor between 2015 and 1979 was dominated by changes in FDsne.

### Environmental factors driving functional diversity

**Figure 4:**
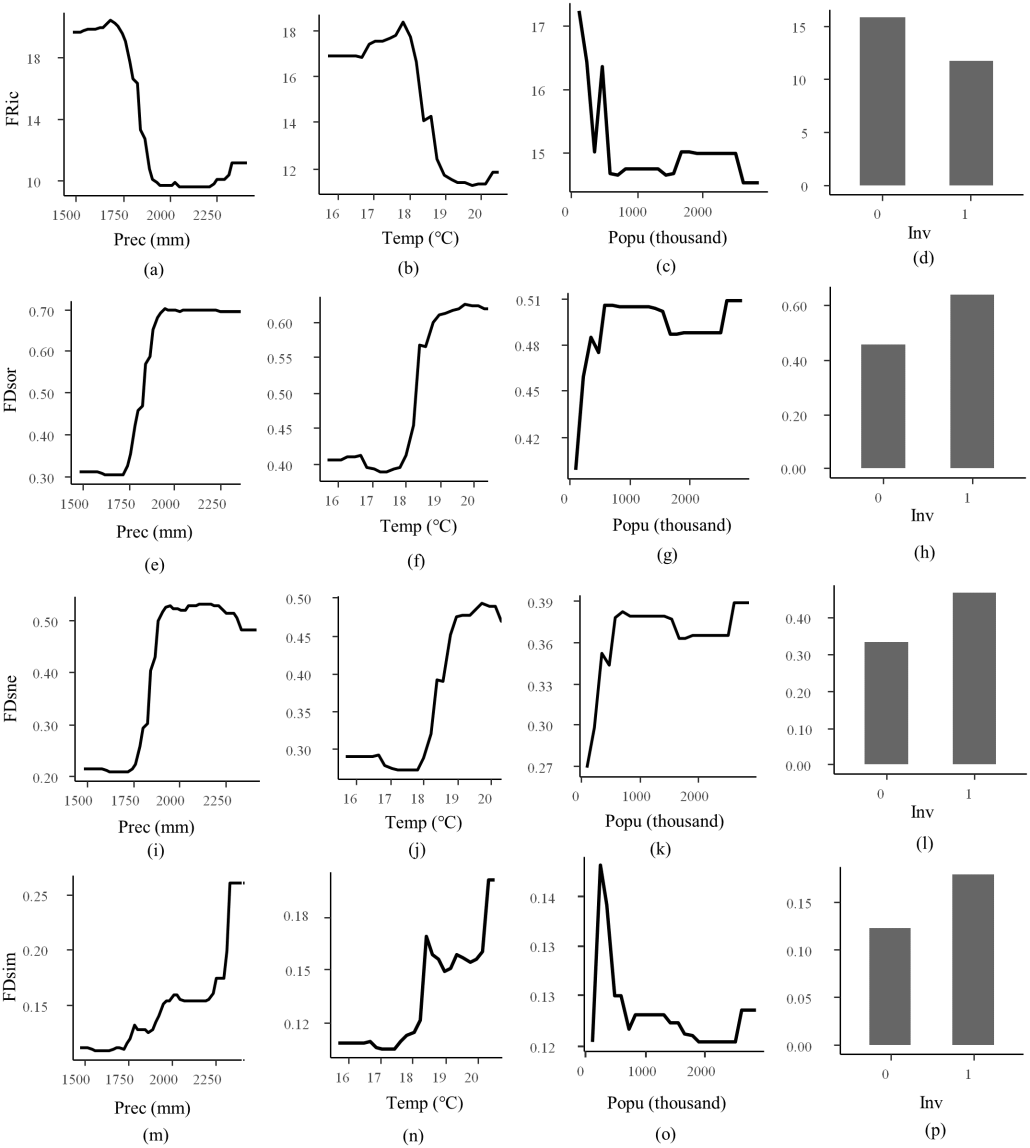
Partial dependence plot for random forest regression. Responsive variables are functional richness (FRic, A–D), total beta Sørensen (FDsor, E–H), nestedness (FDsne, I–L), and turnover (FDsim, M–P). Explanatory variables are precipitation (Prec), temperature (Temp), population (Popu), and species invasion (Inv). Value “1” for Inv refers to introduction of invasive non-native species, and “0” refers to no occurrence of such species.

RF models for FRic, FDsor, FDsne, and FDsim explained 83.83%, 88.78%, 83.42%, and 35.75% of the total variances, respectively (Table 2). Models for FEve and FDiv were not explanatory which explained less than 10% of the total variance. Ranking by their relative importance, precipitation, temperature, species invasion, and human population were the most important factors for modeling FRic, FDsor, FDsim, and FDsne, except that human population was not explanatory for FDsim. Partial dependence plot showed that responses of FRic to precipitation, temperature, species invasion, and human population were negative, while responses of FDsor, FDsim, and FDsne to precipitation, temperature, species invasion, and human population were positive, except that response of FDsim to human population was irregular (Fig. 4).

### Correlations between functional traits and environmental factors

**Figure 5:**
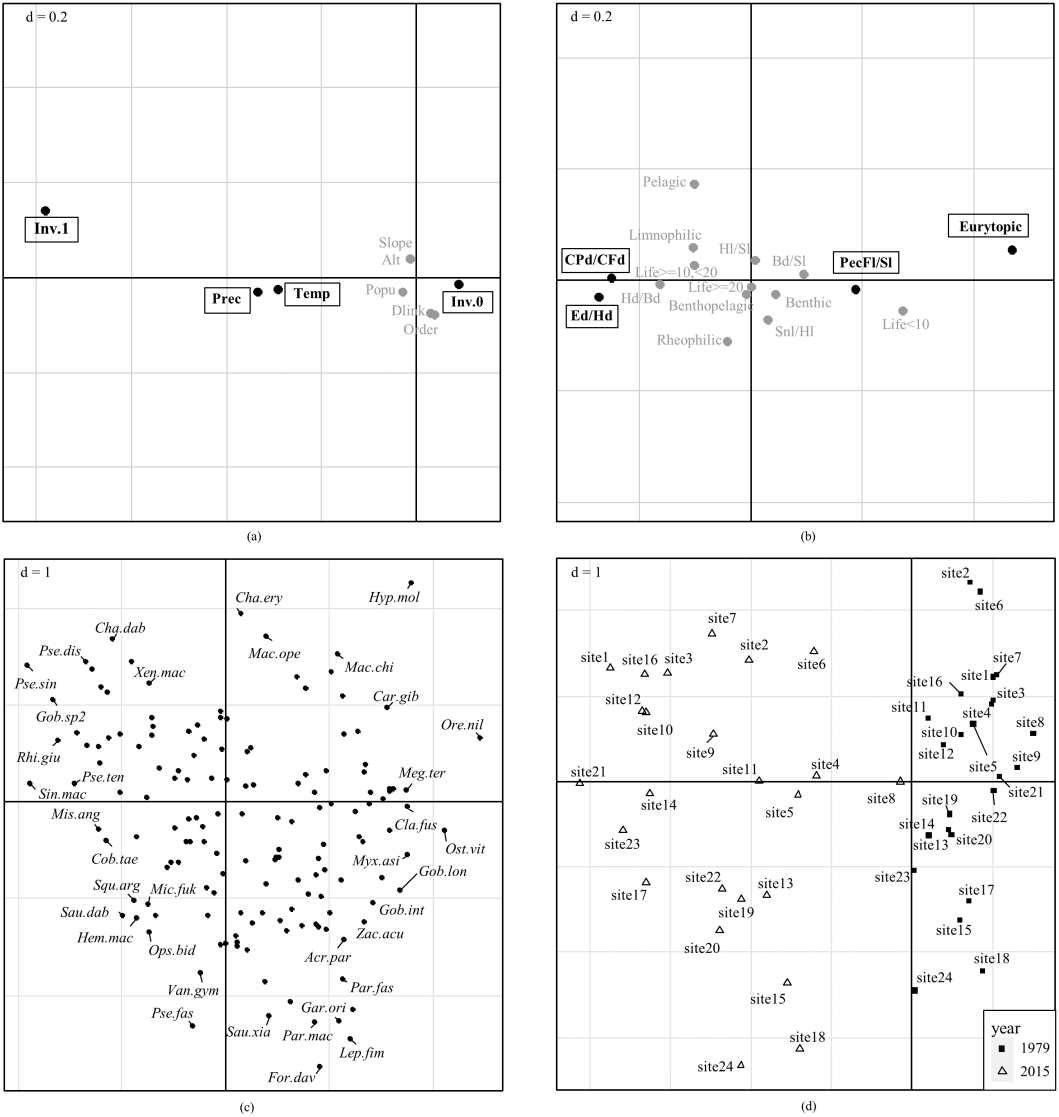
Combination of fourth-corner with RLQ results. Ordinations of (A) environmental factors, (B) fish functional traits, (C) species, and (D) sampling sites are reported on the ordination diagram of RLQ analysis. Bold black text with rectangles in (A) and (B) represent significant association with the first axis, while variables with no significant association are in gray. *P* values were adjusted for multiple comparisons using the false discovery rate (FDR). No significant correlations with the second axis are detected. Variables in (A) are Temperature (Temp), precipitation (Prec), Altitude (Alt), slope (Slope), Population (Popu), Strahler order of stream segment (Order), downstream link (Dlink), presence of invasive species (Inv.1), and absence of invasive species (Inv.0). Codes for fish functional traits in (B) are listed in Table 1, and codes for species names in (C) are shown in Table S2.

Global significance test rejected H_0_ and confirmed that fish functional traits were significantly correlated with environmental factors (*p* <0.05 for both Model 2 and Model 4, Table 3). Axis 1 and Axis 2 of the RLQ analysis explained 90.40% and 6.54% of the total co-inertia, respectively. The first RLQ axis was negatively correlated with precipitation, temperature, and presence of invasive species, and positively correlated with absence of invasive species (Fig. 5A). Significantly associated traits with higher precipitation, higher temperature, and presence of invasive species were higher swimming factor (minimum caudal peduncle depth divided by maximum caudal fin depth), higher relative eye size (eye diameter divided by head depth), and lower relative pectoral fin length (pectoral fin length divided by standard length) (Fig. 5B). Traits associated with absence of invasive species, lower temperature, and lower precipitation were eurytopic water flow preference, higher relative pectoral fin length, lower swimming factor, and lower relative eye size. No significant associations were detected for the second axis, neither for environmental factors nor for traits.

**Table 2:** Summary of the most important environmental factors extracted from random forest modeling. “Var%” indicates percentage of total variance that the random forest model explains. “N/A” indicates an unsuitable RF model whose Var% is below 10%. Decimal digits in other cells are values of the percentage of increased mean standard error (MSE) when corresponding environmental factor is permuted in each model. The more the MSE is increased, the more important this variable is. “NP” means this factor is non-explanatory in the model. “(-)” means the response of corresponding FD index to the factor is negative, and “(+)” means positive. Environmental factors are temperature (Temp), precipitation (Prec), population (Popu), altitude (Alt), slope (Slope), species invasion (Inv), Strahler stream order (Order), and downstream link (Dlink). Responsive variables are functional richness (FRic), functional evenness (FEve), functional divergence (FDiv), total functional beta diversity (FDsor), nestedness (FDsne), and turnover (FDsim)

	**FRic**	**FDsor**	**FDsne**	**FDsim**	**FEve**	**FDiv**
Var%	83.83	88.78	83.42	35.75	*N/A*	*N/A*
Prec	76.65 (-)	78.43 (+)	76.49 (+)	53.85 (+)	*NP*	*NP*
Temp	50.98 (-)	51.12 (+)	50.14 (+)	26.47 (+)	*NP*	*NP*
Inv	40.51 (-)	47.12 (+)	26.76 (+)	25.41 (+)	*NP*	*NP*
Popu	23.65 (-)	18.64 (+)	38.36 (+)	*NP*	*NP*	*NP*
Alt	*NP*	*NP*	*NP*	*NP*	*NP*	*NP*
Slope	*NP*	*NP*	*NP*	*NP*	*NP*	*NP*
Order	*NP*	*NP*	*NP*	*NP*	*NP*	*NP*
Dlink	*NP*	*NP*	*NP*	*NP*	*NP*	*NP*

**Table 3:** Results of the RLQ analysis. The first two axes of RLQ analysis are extracted. Global significance is tested with 9,999 repetitions. Model 2 test the relationship between species occurrence and environmental factors. Model 4 test the relationship between species occurrence and fish functional traits. Null hypothesis that fish functional traits are not related to environmental factors is rejected if both *p* values of these two models are below the significance level of 0.05.

**RLQ analysis**	**Axis 1**	**Axis 2**
Eigenvalues	0.27	0.02
Covariance	0.52	0.14
Correlation	0.30	0.10
Projected inertia (%)	90.40	6.54
Model 2	*p*= 0.001	
Model 4	*p*= 0.001	

Species associated with higher swimming factor and relative eye size were endemic species *Sinibrama macrops*, *Pseudobagrus tenuis*, and *Pseudolaubuca sinensis,* etc. (Fig. 5C). These species occurred in sites with higher precipitation, higher temperature, and presence of invasive species. Species associated with eurytopic water flow preference and higher relative pectoral fin length were nilem carp *Osteochilus vittatus*, *Gobiobotia longibarba*, and catfish *Clarias fuscus,* etc. These species were correlated with the opposite environmental conditions.

Sites in 2015 and 1979 were clearly separated on the ordination diagram (Fig. 5D). Sites in 2015 were characterized by environmental conditions with presence of invasive species, higher precipitation, and higher temperature, and associated with fish species with higher swimming factor, higher relative eye diameter, and lower relative pectoral fin length. Sites in 1979 were characterized by opposite environmental conditions and associated with species with eurytopic water flow preference and higher pectoral fin length. Meantime, sites in 2015 were more disaggregated than in 1979 on the ordination diagram, suggesting that trait compositions in 2015 were more varied than in 1979.

## Discussion

### Temporal changes of functional diversity and community assembly

The Min River ever possessed diverse and distinctive fish species accounted by its heterogeneous microhabitats in history (Zhu, 1984). For example, Chinese sturgeon *Acipenser sinensis* and Chinese sucker *Myxocyprinus asiaticus* dwelled in the river according to historical records (Kang et al., 2014). Chinese sturgeon *A. sinensis* is listed as a critically endangered species on IUCN red list (IUCN, 2020). Although there have been some conservative attempts such as artificial propagation and release since 1980 to protect this species in the Min River (Kang, 2002), no individual was sampled in 2015. Chinese sucker *M. asiaticus* is an endemic species and quite popular in local aquariums in the Min River (Editorial Committee of Fujian Province Annals, 1999). This fish was collected at sites 11, 12, and 13 in 1979, but was absent from all these sites in 2015. Those 69 species that were collected in 1979 but absent in 2015 are all native to the Min River. For example, yellowcheek *Elopichthys bambusa*, long spiky-head carp *Luciobrama microcephalus*, and *Ochetobius elongates* are endemic species with high economic values (Editorial Committee of Fujian Province Annals, 1999). They have become rare nowadays in major rivers and lakes in China due to overfishing and habitat changes (Feng et al., 2014; Zhang et al., 2010).

FRic is known to be positively correlated with species richness (Villeger, Mason & Mouillot, 2008). Loss of native species with distinctive functional traits diminishes the functional diversity and leads to functional homogenization (Chua, Tan & DCJ, 2019). The temporal decrease of FRic in this work was directly linked to the reduction of species richness in 2015 comparing with 1979. Temporally homogeneous and stable habitat supports relatively higher FEve (Massicotte et al., 2014). On the other hand, intense land-use/cover changes under human interferences in some streams have been associated with a decrease in FEve (Barbosa, Pires & Schulz, 2020). Variance of FEve in 2015 was significantly higher than that in 1979, which indicates that fish functional traits in 2015 were distributed more unevenly than in 1979, and this unevenness possibly suggests temporally unstable habitat conditions.

FDsne refers to functional dissimilarity resulted from trait loss (Villeger, Grenouillet & Brosse, 2013). Higher FDsne in 2015 than 1979 reflects that smaller communities became functional subsets of larger ones as a consequence of trait loss. At local scales, ecosystems suffering from intense interferences manifest higher FDsor. For example, high-impact environments show higher FDsor and FDsne but lower FRic in cladoceran communities in Northeastern and Southern Brazil (Simoes et al., 2020). The dominance of FDsne in this work further indicates that loss of distinctive functional traits as a result of species loss might be accountable for the temporal pattern of functional beta diversity.

Species loss is associated with habitat deterioration in many cases, which strengthened environmental filtering. For example, local-scale factors related to habitat degradation were associated with fish trait convergence and decreases of fish species richness in a Mediterranean river (Radinger, Diego Alcaraz-Hernandez & Garcia-Berthou, 2019). FDiv in 2015 was significantly lower than that in 1979, indicating that more trait convergence, i.e., strengthened environmental filtering, occurred in 2015 than in 1979. Patterns in community structure and functional diversity of freshwater fish communities at global scales are correlated with abiotic or biotic filters (Comte et al., 2016). At local scales in the Min River, species loss and increased trait convergence indicate that temporally strengthened environmental filtering was the main temporal community assembly mechanism, which reconciles the fact that the Min River has been exposed to multiple interferences, including climate change, human disturbances, and species invasion.

### Importance of environmental factors driving functional diversity

Global climate change impacts freshwater fish FD worldwide (Buisson et al., 2013). High fish FD loss in Southeast Asia, Indo-Burma, and South-Central China is accompanied by global climate change (Macdonald et al., 2020). Intensity of anthropogenic activities firmly associates with fish FD. For instance, increasing human pressure in Neotropical reservoirs is negatively correlated with fish FD (Dias, De Oliveira & Baumgartner, 2021). Species invasion also undermines regional fish FD by outcompeting local species (Milardi et al., 2019). There are over 400 species of non-native freshwater fish species in China, most of which were introduced exotically by aquarium and aquaculture industries (Xiong et al., 2015). Similar factors have been recognized in the Min River. For example, temperature and precipitation were increased by around 1.5 °C and 390 mm on average in 2015 compared with 1979 in the basin, respectively; human interferences, such as land-use/cover changes under urbanization, dam construction, and agriculture, have been intensified during the past decades (Ma et al., 2020; Ren et al., 2016; Zhao et al., 2016); several invasive species have been identified in the basin (Deng et al., 2020). The synergic effect of these factors might contribute to the loss of habitat suitability for fishes in the Min River.

There has been a growing amount of evidence that climatic change is a strong determinant for fish FD (Kuczynski et al., 2018). Fish habitat suitability is expected to shift under scenarios of global climate change for physiological, geological, or hydrochemical reasons (Marras et al., 2015; Xing et al., 2019). In the Min River, precipitation and temperature were the most influential factors driving the patterns of FRic, FDsor, FDsim, and FDsne. Decadal changes in temperature and precipitation possibly alter fish habitat conditions, including water temperature, duration of flood or drought season, water velocity, and water depth, which influence species distribution (Chang & Jung, 2010). For example, variations of temperature and precipitation in spring and summer changed fish communities in the Lower Colorado River Basin (Pool et al., 2010). This supports the result as previously discussed that environmental filtering is the dominant mechanism driving fish community assembly in the Min River temporally.

Several invasive species were collected in 2015, which occurred at 11 out of 24 sites in the river. While in 1979, none of these species were collected at these sites. These species are grass carp *Ctenopharyngodon idella*, Wuchang bream *Megalobrama amblycephala*, Mozambique tilapia *Oreochromis mossambicus*, Chinese catfish *Hemibagrus macropterus*, warmouth *Lepomis gulosus,* and Chinese loach *Sinibotia superciliaris* (Deng et al., 2020). For example, invasive species like Mozambique cichlid *O. mossambicus* occurred at site 1, site 3, and site 10, and meantime grass carp *C. idella* at site 10. Mozambique cichlid *O. mossambicus* is a successful and vagile invader. Grass carp *C. idella*, which originated from the Yangtze River in China, was a pest because of its damage to submerged vegetation (Deng et al., 2020). Introduction of these species possibly excludes endemic species with similar niches. For example, endemic species mud carp *Cirrhinus molitorella*, a near-threatened fish on IUCN red list (IUCN, 2020), was collected at sites 1 and 10 in 1979 but was absent in 2015. This species grazes on algae, phytoplankton, and detritus, which is a similar dietary with Mozambique cichlid *O. mossambicus* (Froese & Pauly, 2019). The importance of species invasion revealed by RF models indicates that these negative biotic interactions also contribute to community structure in the Min River.

Human population was the least important factor which was explanatory for FRic, FDsor, FDsne, and FDsim. Many anthropogenic activities associate with human population. Urbanization stimulates natural resource consumption and freshwater supplies for cities. Increased demand for water supply indicates more water reservoirs to build, and water pollution is aggravated in areas with high human population density (De Mello et al., 2020). People’s demand for food is also influential. For example, citizens prefer certain fishes such as yellow catfish *Tachysurus fulvidraco* in local cuisine (He et al., 2012). Human population along the Min River had increased by 36% from 1979 to 2015, which indicates strengthened temporal human influences. Nevertheless, ranking the relative importance of different factors confirms that environmental filtering is the dominant mechanism driving fish community assembly in the Min River temporally.

### Responses of functional traits to environmental variables

Ordination of environmental factors and fish functional traits based on fourth-corner combined with RLQ supports previous findings that precipitation, temperature, and species invasion were the most important factors driving fish functional diversity. Meantime, sites in 2015 were more disaggregated than in 1979 on the ordination diagram, suggesting that trait compositions in 2015 were more varied than in 1979. This supports the previous finding that functional beta diversity in 2015 was significantly higher than that in 1979.

Higher swimming factor and higher relative eye size were significantly correlated with higher precipitation, higher temperature, and presence of invasive species. Swimming factor is directly associated with fish swimming capability. Higher values of swimming factor indicate relatively higher water propulsion per swing of the caudal fin (Villeger, Grenouillet & Brosse, 2013). This possibly requires that fish species living in habitats with higher precipitation and temperature adapt to fast-flowing water. For example, endemic species *Pseudobagrus tenuis* and *Pseudolaubuca sinensis* with higher swimming factor and relative eye diameter both occurred at sites 3 and 10 with a relatively higher average temperature of 19 °C and annual precipitation of about 2,000 mm. These two sites were also inhabited by invasive omnivorous species Mozambique cichlid *O. mossambicus*, which possibly incurred intensified interspecific competition for food with *P. tenuis* and *P. sinensis*. Meanwhile, relative eye diameter is correlated with the ability for food detection (Beston et al., 2019). Higher swimming capability and relative eye diameter of these two species assure that they can secure adequate food resources successfully in an ecosystem with fast-flowing water and intense interspecific competition (Starrs et al., 2017).

Eurytopic water flow preference and higher relative pectoral fin length were significantly correlated with absence of invasive species, lower precipitation, and lower temperature. Pectoral fin length is assumed to be correlated with the capability of low-speed maneuvering of fishes (Jones et al., 2020; Watson & Balon, 1984). For example, endemic species *Gobiobotia longibarba* has a higher relative pectoral fin length. It was collected at sites 12, 13, and 14 in 1979 but absent in 2015. Annual precipitation at these three sites was about 1,680 mm in 1979 but was 2040 mm in 2015. Lower precipitation indicates lower water velocity during the flood season. Endemic species like *G. longibarba* thus do not need to resist fast-flowing water nor to frequently swim fast to compete for food resources. On the other hand, higher relative pectoral fin length gives these fishes higher slow-speed maneuvering performance in relatively slow-flowing water, which is possibly important to detect food in local habitats (Jones et al., 2020).

## Conclusions

This work reveals that environmental filtering is the dominant temporal assembly rule in a typical freshwater riverine ecosystem in subtropical areas in China. Species loss is a threat to fish functional diversity, and fish trait structures are shaped temporally through the interactions between fish traits and environmental factors, including temperature, precipitation, and species invasion. Results of this work can help predict biodiversity redistribution under climate change and inform freshwater fish conservation.

##  Supplemental Information

10.7717/peerj.11824/supp-1 Supplemental Information 1 Raw data of species occurrence in the 1970s

10.7717/peerj.11824/supp-2 Supplemental Information 2 Raw data of species occurrence 2015

10.7717/peerj.11824/supp-3 Supplemental Information 3 Raw data of fish traits in the 1970s

10.7717/peerj.11824/supp-4 Supplemental Information 4 Raw data of fish traits in 2015

10.7717/peerj.11824/supp-5 Supplemental Information 5 Basic morphological traits measurement for fishes in the Min River Sl: Standard length; Hl: Head length; PecFl: Pectoral fin length; Snl: Snout length; Bd: Body depth; Hd: Head depth; Ed: Eye diameter; CPd, Caudal peduncle depth; CFd: Caudal fin depth.

10.7717/peerj.11824/supp-6 Supplemental Information 6 Species richness at different sites in 1979 and 2015 SR indicates species richness, FRic refers to functional richness, mean (null) and sd (null)** is the mean value and standard error of FRic extracted from null models, SES is standardized effect size of FRic, and *p* values is extracted from t test.

10.7717/peerj.11824/supp-7 Supplemental Information 7 Codes for species in the Min River

## Additional Information and Declarations

### Field Study Permissions

The following information was supplied relating to field study approvals (i.e., approving body and any reference numbers): Field experiments were approved by the Research Council of Ocean University of China (No. 20150305004).

### Data Availability

The following information was supplied regarding data availability: The raw fish occurrences data and raw fish functional traits are available in the Supplemental Files.
