# Supplementary figures and images for: How fish traits and functional diversity respond to environmental changes and species invasion in the largest river in Southeastern China
Source: PeerJ. 2021 Jul 23;9:e11824. doi: 10.7717/peerj.11824 (PMC8312501; doi:10.7717/peerj.11824)

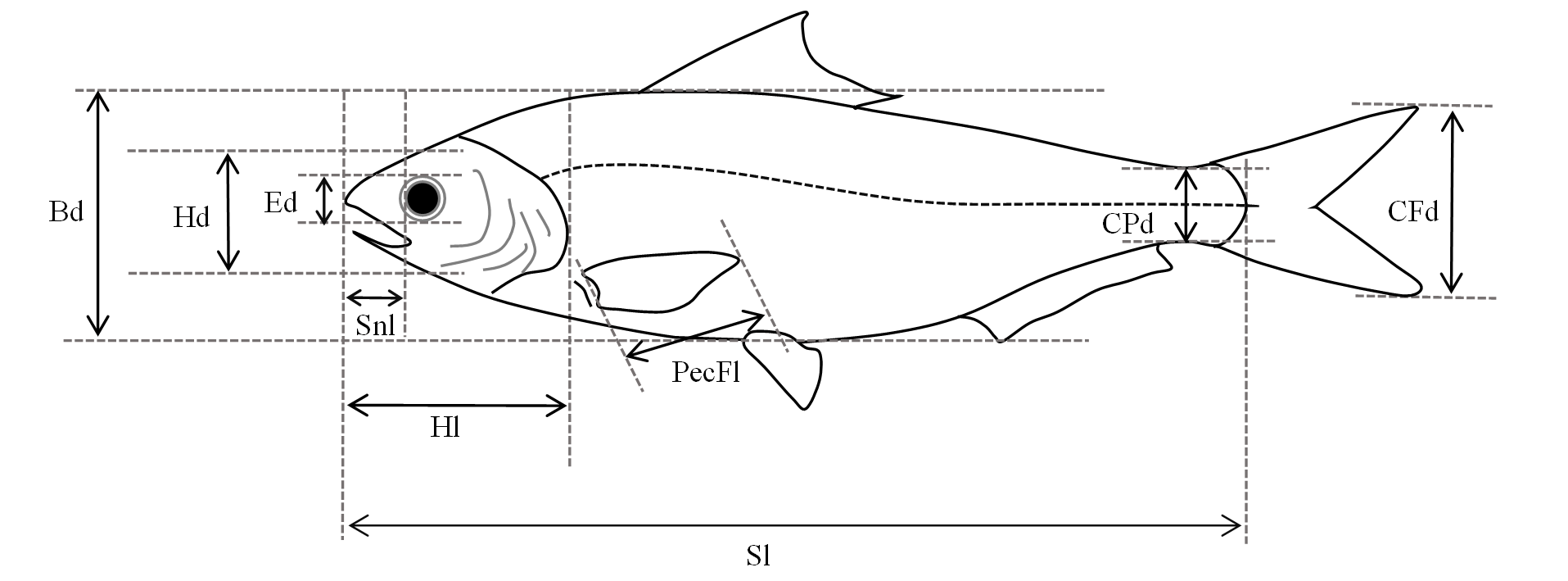

Supplement: Supplemental Information 5 — Sl: Standard length; Hl: Head length; PecFl: Pectoral fin length; Snl: Snout length; Bd: Body depth; Hd: Head depth; Ed: Eye diameter; CPd, Caudal peduncle depth; CFd: Caudal fin depth. [file peerj-09-11824-s005.png]
