# Supplementary material for: How fish traits and functional diversity respond to environmental changes and species invasion in the largest river in Southeastern China
Source: PeerJ. 2021 Jul 23;9:e11824. doi: 10.7717/peerj.11824 (PMC8312501; doi:10.7717/peerj.11824)
Supplement: Supplemental Information 6 — SR indicates species richness, FRic refers to functional richness, mean (null) and sd (null) is the mean value and standard error of FRic extracted from null models, SES is standardized effect size of FRic, and p values is extracted from t test. [file peerj-09-11824-s006.docx]

| Site | 2015 | 1979 |
| --- | --- | --- |
| site01 | 16 | 34 |
| site02 | 7 | 56 |
| site03 | 10 | 39 |
| site04 | 9 | 54 |
| site05 | 17 | 54 |
| site06 | 8 | 39 |
| site07 | 16 | 36 |
| site08 | 10 | 36 |
| site09 | 6 | 45 |
| site10 | 12 | 54 |
| site11 | 10 | 77 |
| site12 | 9 | 76 |
| site13 | 7 | 79 |
| site14 | 12 | 71 |
| site15 | 11 | 73 |
| site16 | 18 | 56 |
| site17 | 7 | 58 |
| site18 | 11 | 45 |
| site19 | 17 | 66 |
| site20 | 10 | 34 |
| site21 | 35 | 33 |
| site22 | 27 | 41 |
| site23 | 13 | 33 |
| site24 | 11 | 33 |
